# Adult Attachment Representations and Body Image

**DOI:** 10.3389/fpsyg.2021.724329

**Published:** 2021-09-10

**Authors:** Nikolay Bonev, Vanya Matanova

**Affiliations:** Master Program in Clinical Psychology, Department of Social, Work, Clinical and Educational Psychology, Faculty of Philosophy, Sofia University “St. Kliment Ohridski”, Sofia, Bulgaria

**Keywords:** attachment, attachment disorganization, body image, representations, trauma

## Abstract

In modern literature, the body image is interpreted as a multidimensional construct, which is considered important for both individual development and quality of life. The body image is central to the self-concept and has important consequences for mental functioning. A negative body image can result in adverse psychosocial consequences for both sexes. For a long time in the professional literature the study of socio-cultural factors on the development of the body image has prevailed. This line of research creates social constructivism, in which the earliest attachment relations are eliminated, and instead the idea is suggested that external sources have a direct influence. The present text proposes an approach to the body image as a development construct, arising and developing in the attachment relations, related to the provision of security and protection. Attachment disturbances, as well as attachment disorganization, are defined as the inability to provide security and protection. Attachment relationships in connection with the development of body image develop through the mechanisms of reflection, sensitive responses to the child’s signals and synchronous relationships. In the first months of human life, the attachment needs are first and foremost the needs of the body, which are satisfied by the responses of the primary caregiver. The topic of body image discusses attachment disorganization, the understanding of attachment trauma, and the “enactment” of the loss on the body’s territory. Trauma always involves loss. Griefs that cannot be mourned and injuries that cannot be represented seem to be central to understanding the body’s problematization.

## Introduction

In the modern literature, the concept of body image is perceived as a multidimensional construct, which refers to: (1) the individual’s perceptions of their body; (2) the disposition/attitudes toward these perceptions; and (3) visible behaviors in response to these perceptions (Cash and Labarge, 1996; Cash, 2004a; Grogan, 2008). Body image can be thought of as individual experiences of the physical self (Cash, 2004a). There are two main aspects that include attitudes toward the body self-image evaluation and investment. Affect refers to the emotions associated with body image in specific situations. The body image has long been considered important for both individual development and quality of life, although it represents only one aspect of self-conceptualization (Sandoz and Wilson, 2006). The body image is central to the concept of the self and has important consequences for mental functioning. A negative body image can result in adverse psychosocial consequences for both sexes – eating disorders, depression, social anxiety, sexual dysfunction, suicidal tendencies, low self-esteem, and low quality of life (Cash, 2004b; Cash et al., 2007; Crow et al., 2008; Zaitsoff and Taylor, 2009). High levels of body dissatisfaction and weight concerns are common in adulthood. Dissatisfaction with one’s body, eating disorders, and extreme methods of weight control are strong risk factors for the development of eating disorders (Guti rrez-Maldonado et al., 2010). Bruch (1962) also noted that a dysfunctional experience of the body image is a central aspect of anorexia nervosa. Body image disorders are now considered a key element in eating disorders and are part of the criteria for diagnosing anorexia and bulimia nervosa.

In recent decades, research in the professional literature has been based on the understanding of the development of body image, determined primarily by the influence of social factors. Thus, for a long time in the literature, the study of the influence of sociocultural factors on the formation of the body image has prevailed, which has led to social constructivism, in which the earliest attachment relations are eliminated, and substituted with the idea that external sources have direct influence. The body image should also be considered in the context of internal representations and the set of fantasies and meanings and understandings of the body, its parts, and functions (Krueger, 1988). In this sense, the image of the body can be thought of as a dynamically and developmentally emerging mental representation of the bodily self. The bodily self and body image are developmental processes that undergo gradual changes associated with maturation (Krueger, 2004).

## Discussion

The mother defines her baby’s body in the systemic and relationship-based matrix of attachment – providing care. This definition of the body also occurs in the tactile delineation, reflection, and resonance of the internal and external bodily experiences. The theory of attachment is considered as part of the theories of object relations. The shared understanding in these theories is that the basic emotional motivation in human life is the desire to form and maintain relationships with others. The purpose of these relationships is related to survival, the pleasure, and satisfaction that these significant others can provide (Steele and Steele, 2005). One of the main goals of John Bowlby in formulating the theory of attachment was to create a new model of developmental psychopathology that emphasizes the role of real-life events as relevant to some aspects of personal development and mental health (George et al., 1999; Matanova, 2015a, b). In his view, starting from infancy, individuals construct representational models of attachment (internal working models – IWM), which are based on their real relationship with the figure of attachment (George and West, 2001). Bowlby’s understanding focuses on the ideas of environmental failure and traumatic experiences related to the impossibility of *providing security and protection*.

We approach the development and violation of the image of our own body in a new way. The topic of body image is not new at all, but we believe that the approach we take to it is new. We consider the concept of body image as a developmental construct, arising and developing in relations with significant others. The development of the body and the image of it are mostly object-related. The approach we have taken in discussing the topic refers and is limited to the relations of attachment – their development and violation. Disorders of attachment, as well as its disorganization, are defined as the impossibility of providing security and protection. Relationships of attachment in connection with the development of body image develop through the mechanisms of reflection, sensitive responses to the child’s signals, and synchronous relationships. All these interactions are accepted to be the foundation of security.

Numerous studies discuss adult attachment style and attitudes toward the bodily self, but these studies do not belong to the developmental understanding of disorders of body attitudes. They do not clarify the question – whether the style (attachment style vs. attachment representation) of attachment is the cause of disorders in body image. The results emphasize the social value of understanding the image of the body in relation to the processes of *social attachment*. For their part, feelings about one’s own body reflect developmental attitudes more than the styles of social and romantic attachment adopted by the self.

The early experience of security in attachment relationships is critical to the mental integration of traumatic events (Solomon and George, 1996; George and Solomon, 2008). This internalization of the experienced security in the relationship allows the individual to build an inner safe shelter for themselves. Insecure attachment (avoidant and ambivalent) stimulates the development of a “false bodily self” (Lemma, 2015). In the first months of human life, the needs of attachment are first and foremost the needs of the body, which are satisfied by the responses of the primary caregiver. These relationships, according to Krueger (1988), organize and give meaning to the bodily self. Relationships of attachment and subsequent models (attachment patterns) are the primary and central basis from which the physical and mental selves develop and integrate.

Children who are classified as anxious (ambivalent or avoidant) often have mothers who show difficulty and resistance to establishing close physical contact. Children with a secure attachment to their mothers actively seek physical comfort from them, after a short but stressful separation, unlike avoiding children.

The topic of body image discusses the disorganization of attachment, the understanding of the trauma of attachment, and the “playing out” of the loss on body territory. George and West (2012) attribute the trauma of attachment to all experiences that instinctively signal a threat to break the attachment relationship or signal a danger to the self. Irreversible separation, according to Bowlby (1980), is loss as a manifestation of the trauma of attachment. From an evolutionary perspective, for both children and adults, restoring intimacy with the figure of attachment is the only solution to relieving fear. In this sense, the losses – real or symbolic, which cannot be mourned and the traumas, which cannot be represented, seem to be central to the understanding of the body’s problematization (eating disorders; voluntary cosmetic or esthetic surgery). Trauma always involves loss (George and West, 2012). These losses may be too real and literal, such as the loss of a loved one, for example, or they may be more symbolic, such as the loss of identity, meaning, or hope (Lemma and Levy, 2004; Lemma, 2015). A traumatic experience affects not only the ability to connect, to feel, but also the ability to think symbolically. The lack of symbolic functioning leads to a reduction in the ability of the self to know and think about itself, as a separate agent, as an agency of self. This makes it more likely that the body will be used to express what cannot be represented.

In a preliminary study (Bonev and Matanova, Unpublished) the data show that disorganized people compared to organized (safe or insecure) experience more anxiety about their body and are more likely in a consultative and therapeutic context to problematize their body (e.g., dissatisfaction with appearance; symptomatology of eating disorder, modification of the body surface – cosmetic surgery). People who invest more (psychologically) in their appearance, are anxious about gaining weight and are sensitive to the presence of various physical symptoms are more likely to have had caregivers in their history who could not provide them with the experience of sufficient security. Relationship security implies lower levels of anxiety about weight and less dysfunctional behaviors, such as restricting eating and dieting. The specifics of the relationship of attachment are related to the development of attitudes toward the bodily self and its image.

Those who problematize their bodies at the level of appearance, weight, investment in a healthy lifestyle, suspicion of the presence of physical symptoms, have not experienced a relationship with the primary caregiver which could provide sufficient relief of physical and mental sensations and allow them to process the affect and thus to desomatize it. In this sense, it seems that the lack of sensitivity, spontaneity, responsiveness, and pleasure in the relationship of attachment leads to a stronger investment in the bodily self. These elements of sensitivity, spontaneity, and responsiveness underlie the development of mentalization, in which the representation of affect also participates.

When this representation of affect is possible, a memory trace is formed in the baby’s brain that connects the bodily experience with the image of the emotion transmitted by the mother, forming a mental representation of the emotion corresponding to the bodily experience. When there is no representation, then the affect from the experience cannot be desomatized and is unloaded or communicated through the body.

It also seems that the experience of fear associated with attachment, which is the basis of disorganization, is related to the more tangible problematization of the body. According to Bonev and Matanova (Unpublished), the disorders are to some extent related to the behavioral dimension of the body image, but these behaviors in the context of the bodily self are compensatory, aimed at overcoming feelings of anger, fear, vulnerability, inadequacy, or helplessness. Except in the context of “failed protection,” bodily self-disorders in disorganized people seem to be related to the inability to achieve effectiveness in early childhood (around 4 months of age) – the behavioral dimension “capacity to act” (George and West, 2012). These assumptions are based on existing data (George and West, 2001, George and West, 2011, 2012), in which disorganized people cannot cope with the resulting dysregulation in any way. One of the possibilities for resolving (coping with) dysregulation is the activation of the capacity to act, which allows a person to make a constructive change or to move away from the place of dysregulation, to protect her/himself. The basis for the development of this capacity is precisely this experience of the effectiveness of actions, i.e., this is the pleasure of the effect – the pleasure of being the cause (the agent of the action), and not so much with the actual result of the action.

Disorganization of attachment is a violation of the ability to construct dialogue – internal and external, to share experiences with “another who can tolerate and retain what is shared” (Lemma and Levy, 2004). This impossibility of secondary representation of experiences makes it possible for the body to express what there are no words for. In this regard, disorganized people problematize their body in several dimensions. These dimensions are associated with concern for the health of the body; anxiety about weight gain and subsequent dieting; behaviors controlling health and behaviors aimed at improving appearance.

The study of dyadic relations makes it possible to study the processes of projection and introjection that form the self. It is known that the mother’s face is the first emotional mirror for the child (Winnicott, 1967; Lacan et al., 1977) and what it reflects is the “reality for the child” (Krueger, 1988). In this way, reciprocity and synchronicity in relationships seem central to understanding body worries. In cases where the relationship is based on traumatic connection and experiences of rejection, there is emotional sterility in which there is no room for experiences and emotions. The lack of synchronicity between the signals given (by the child) and the answers to them (from the figure of attachment) define the care in these relations as basic and functional, the reciprocity and the pleasure of the relationship are missing, which hinders the representational integration of experiences, which in turn violates recycling capacity.

Appearance orientation distinguishes organized from disorganized attachment groups because investments in appearance represent an attempt at “representational reunion” (Bonev and Matanova, Unpublished) and are an attempt to achieve synchronicity and reciprocity in the representational world. These behaviors are a way to become a desired and loved object. To create an “ideal appearance that will guarantee the love and desire of the other” at least in your own mind (m/other) (Lemma, 2010).

This topic is extremely important because we are embodied living organisms and the development of an internal safe basis begins with the body’s contact with the other’s body, which can provide security and containment of all early and threatening experiences. Because development – physical and mental, is an inseparable connection, which literally means learning how to move through life. The complex system, collectively known as the body, is an expression of the security with which we walk through the world (Shafir, 2018). And the way this body is invested, in the earliest moments of life, will determine the way we will move in the world – mentally, physically, and spiritually.

## Author Contributions

Both authors listed have made a substantial, direct and intellectual contribution to the work, and approved it for publication.

## Conflict of Interest

The authors declare that the research was conducted in the absence of any commercial or financial relationships that could be construed as a potential conflict of interest.

## Publisher’s Note

All claims expressed in this article are solely those of the authors and do not necessarily represent those of their affiliated organizations, or those of the publisher, the editors and the reviewers. Any product that may be evaluated in this article, or claim that may be made by its manufacturer, is not guaranteed or endorsed by the publisher.
